# The Relationship between Dynamic Balance and Jumping Tests among Adolescent Amateur Rugby Players. A Preliminary Study

**DOI:** 10.3390/ijerph18010312

**Published:** 2021-01-04

**Authors:** Bartosz Wilczyński, Jakub Hinca, Daniel Ślęzak, Katarzyna Zorena

**Affiliations:** 1 Department of Immunobiology and Environment Microbiology, Medical University of Gdańsk, Dębinki 7, 80-211 Gdańsk, Poland; kzorena@gumed.edu.pl; 2 Department of Physical Culture, Physiotherapy, Gdansk University of Physical Education and Sport, 80-336 Gdańsk, Poland; jakubhinca@wp.pl; 3 Departament of Medical Rescue, Medical University of Gdańsk, Dębinki 7, 80-211 Gdańsk, Poland; daniel.slezak@gumed.edu.pl

**Keywords:** team sport performance, injury risk screening, athletes assessment, landing error score system, counter movement jump

## Abstract

Rugby is a demanding contact sport. In light of research, poor balance, reduced jumping ability, muscle strength, and incorrect landing patterns might contribute to the increased risk of injury in athletes. Investigating the relationship between tests assessing these abilities might not only allow for the skillful programming of preventive training but also helps in assessing the risk of injury to athletes. Thus, the main purpose of this study was to investigate the relationship between dynamic balance, vertical and horizontal jumps, and jump-landings movement patterns. Thirty-one healthy amateur adolescent rugby players (age: 14.3 ± 1.6 years, height 171.4 ± 9.7 cm, body mass 80 ± 26 kg) participated in the study. Data were collected by the Y-balance Test (YBT), Counter Movement Jump (CMJ), Single Leg Hop for Distance (SLHD), and Landing Error Score System (LESS). Significant positive correlations were found between SLHD both legs (SLHDb) and YBT Composite both legs (COMb) (r = 0.51, *p* = 0.0037) and between SLHDb and CMJ (r = 0.72, *p* < 0.0001). A relationship was also observed between the CMJ and YBT COMb test (r = 0.51, *p* = 0.006). Moderate positive correlations were found between the dominant legs in SLHD and the posterolateral (r = 0.40, *p* = 0.027), posteromedial (r = 0.43, *p* = 0.014), and composite (r = 0.48, *p* = 0.006) directions of the YBT. These results indicate that variables that are dependent on each other can support in the assessment of injury-risk and in enhancing sports performance of young athletes.

## 1. Introduction

Rugby union is a demanding team sport in which players must undoubtedly have adequate physical attributes. During matches and training, players perform many activities such as acceleration, jumping and landing, changing the direction of running, and maintaining body balance in various planes [1,2,3]. Rugby players should have the qualities of strength, power, acceleration, and speed, as well as the appropriate stability and balance [4]. There is a general acceptance that due to the specificity of this sport, the participants are exposed to musculoskeletal injuries [5]. In a prospective cohort study by Haseler et al., 2010, regarding injuries among young English rugby players, an overall injury rate of 24/1000 h player was found [5]. Although injuries in youth rugby are rarer and less severe than in adult rugby, the risk of injury increases with age [5]. Moreover, limitation of balance and strength in the lower limbs are described as significant internal risk factors for injury among adolescents [6,7]. Therefore, it is important to test these factors in athletes and to investigate the relationship between them. These are challenges for trainers, sports scientists, physiotherapists, and strength and conditioning practitioners, to skillfully manage the development of adolescent rugby players.

Dynamic balance as one of the most important skills in rugby is defined as the ability to perform a task while maintaining a stable posture, most often using a single leg support base [4,8]. A popular, reliable, easy-to-use balance assessment tool is the Y-Balance Test (YBT) [9]. Studies demonstrated a link between the YBT score and potential lower limb injuries in various populations [4,10,11]. Importantly, the study by Johnston et al. 2019 revealed that the worse dynamic balance measured by the YBT among rugby union players increases the relative-risk, sports-related concussion [12]. Research supports the thesis that control of the dynamic balance might be affected by some features of neuromuscular performance, such as lower extremities strength [13], core stability [14], and range of motion [15].

Furthermore, a deficit in muscle strength and power is considered as a risk factor for injury among adolescents [6]. A common method for the evaluation of the explosive power of the lower limbs is the Counter Movement Jump (CMJ), where the maximum vertical jump is assessed [16]. Moreover, to assess the horizontal jump and explosive power of a single leg, the single leg hop for distance (SLHD) test is often used [17]. According to a study by Goosens et al., 2015 lower results in the SLHD test might be a risk factor for hamstring injuries [18].

Several publications examined the relationship between balance and hop performance tests for the power of lower limbs, however, current results are inconsistent [19,20]. Erkmen et al., 2010 present a significant correlation between the single-leg balance and vertical jumping and the relationship between the double-leg jump and the total balance score in young adult football players. The researchers concluded that activities that require lower limb power might reflect the ability to stabilize the body posture [19]. In contrast, Granacher and Gollhofer 2011 found no relationship between vertical jumping and static/reactive balance among adolescent students [20]. Another possibly intrinsic injury-risk factor is the incorrect patterns of movement during jump-landing tasks. A commonly used test among trainers and researchers to evaluate the jump-landing pattern is the Landing Error Score System (LESS) [21,22]. Participants with a lower score (higher score) demonstrate jump-landing technique errors in the frontal and sagittal plane [21]. For example, youth soccer players with poor landing patterns are more likely to sustain injuries [21,22].

The relationship between the power and balance of the lower extremities might be due to similar neuropsychological structures responsible for controlling the posture and power of lower extremities. The same information path (from Ia afferents) acting on the motor neuron is responsible for the production of muscle power and maintaining balance. Moreover, both voluntary muscle activity and the control of long latency reflex during balance tasks are driven by cortical excitability [23,24]. In addition, mechanoreceptors located in the muscles (muscle spindles) and the tendons (Golgi tendon organ) that perform reflex functions support the positioning (e.g., axial) of the lower limbs during movement tasks [25].

The use of tests to assess the dynamic balance, jumping abilities and landing patterns as injury risk factors seem to be justified in terms of injury prevention in athletes. To effectively support the development of interventions that reduce the risk of injury, it is important to know the association between the assessed neuromuscular abilities [23,26]. Therefore, this study aimed to determine the relationship between dynamic balance and jumping tests in adolescent male rugby players. The hypothesis assumed a significant relationship between these variables, which could help in assessing the risk of injuries and in designing preventive interventions and improving sports performance in young athletes.

## 2. Materials and Methods

### 2.1. Participants

The study involved 31 healthy adolescent male (age: 14.3 ± 1.6 years, height 171.4 ± 9.7 cm, body mass 80 ± 26 kg) amateur rugby players from a rugby club in Poland. Participants were excluded when they had an injury or limitation preventing them from playing sports and implementing a test procedure for the purposes of the study.

The subjects and their parents/guardians were informed and gave their written consent for the participation of their children in the study. The study was approved by the Independent Bioethical Committee for Scientific Research at the Gdańsk Medical University (resolution NKBBN/697/2019-2020/).

### 2.2. Procedures

All tests were carried out at the premises of the National Rugby Stadium in Gdynia, Poland. 

The tests took place over three days, free of training and matches (before the start of the league games, in the afternoon, from 12:00 to 5:00 p.m.) in February 2020, in a sports hall at room temperature in the rugby training complex. Before starting each test, the participants obtained the necessary information and a demonstration of the test, correctly performed by an experienced physiotherapist and performance trainer. Basic anthropometric data (body mass, BMI, height) were collected from a body composition analyzer (InBody 270, InBody Co., Seoul, Korea). The author’s questionnaire allowed obtaining data regarding the dominant leg, position on the field, training experience (Table 1).

#### 2.2.1. Dynamic Balance

The Y-Balance Test kit (Move2Perform, Evansville, IN, USA) was used to measure the quantitative values of lower limb dynamic balance [9]. Participants standing single-legged on the platform moved the blocks as far as they could, with their free limb, in the direction of the anterior (ANT), posterolateral (PL), and posteromedial (PM). The test was performed with procedures and instructions for standardization, following the protocol of Plisky et al., 2008 (video and verbal instructions before the start, participants are without shoes, 6 practice tests in each direction to minimize the learning effect). The tests were repeated if the participant lost his balance, leaned on the ground with his foot, kicked the platform, or raised his heels while moving the block [9,27]. The test consisted of 3 correct attempts for the left and right lower limbs. An experienced tester evaluated the sample for errors and recorded the result in centimeters, for each direction. Then, the participants were evaluated for lower limb length in a supine position (from the anterior superior iliac spine to medial tibial malleolus).

The maximum distance of each direction for the dominant and non-dominant leg was used for the analysis, which was normalized to the length of the lower limbs, divided by the length of the lower limbs and then multiplied by 100 (LL%). Composite (COM) reach distance for each lower limb was calculated as the sum of 3 directions divided by 3 times the length of the lower limbs and multiplied by 100 [9]. COMb (Composite—both legs) was defined as the average of the COM results of the dominant and non-dominant legs. YBT indicated good interrater and intrarater reliability in previous studies [9,27].

#### 2.2.2. Countermovement Jump (CMJ)

The countermovement jump (CMJ) was used to investigate the explosive power of the lower extremities [16]. The study participants were requested to stand with both feet on a contact mat (Fusion Sport Smart Jump mat, Fusion Sport, 2 Henley ST, Coopers Plains, QLD, 4108, Australia). The subjects were instructed to keep their hands on their hips (for controlling arm contribution), before and during the jump [28]. The countermovement jump was established as the preferred self-selected depth position in previous research [29,30]. The correct attempt was when the participant had straightened his knee joints during flight and initial landing contact. There was a 2-min break between jumps [13]. Highest jump (cm) from three maximal attempts were selected for data analysis [28]. CMJ is a reliable and valid flight-time-based method that allowed the assessment of the maximal vertical jump height [16].

#### 2.2.3. Single Leg Hop for Distance (SLHD)

Subjects standing on one leg with hands resting on hips in front of the starting line. Immediately after the tester’s signal, they made a forward leap as far as they could and landed on the same leg. The tests were performed alternately for the left and right legs, with a 30-s break between tests. The attempt was correct when the participant kept his balance without supporting himself with another limb for at least 2 s. The distance obtained was measured in centimeters. The best result of 3 jumps for the dominant and non-dominant leg was taken for analysis. SLHDb (SLHD—both legs) was defined as the average of the maximum jumps of the dominant and non-dominant lower limb [31]. SLHD exhibited excellent test–retest reliability, in a recent study [17].

#### 2.2.4. Landing Error Score System (LESS)

LESS is a clinical assessment tool for finding incorrect movement patterns (errors). Participants make a horizontal double-leg jump from a 30 cm box beyond the designated line (distance 50% of the participant’s height). Immediately after landing, they made the maximum vertical jump. Several (typically 3) practice jumps were allowed to perform the task successfully. Then, the participants performed 3 jump-landing tasks with a 2-min break between them. Two video cameras (GoProHero 4, GoPro, Inc., San Mateo, CA, USA) were set up in front and to the side, 3 m from the participant performing the jump-landing task [21,22].

Recording from the camera allowed an accurate assessment of the movement patterns (errors) to be made, and determination of the score. The LESS tool contains 17 sections to assess the characteristics of the landing in the frontal and sagittal planes. A larger result suggests a worse technique and more landing pattern errors. On the same day, an experienced physiotherapist who did not take part in the study assessed the record of jumps-landings from cameras, using the Kinovea^®^ program (beta-version 0.8.26, Bordeaux, France). From the three samples, the best (smallest result) was taken for analysis.

### 2.3. Statistical Analysis

All variables were examined by the Shapiro–Wilk distribution normality test. Means and standard deviations were calculated for all variables. Pearson correlation coefficients were calculated between the CMJ, LESS, SLHD, and Y-Balance Test. To determine the strength, the correlation coefficient was used (r): strong (0.50 ≤ r ≤ 1.0), moderate (0.3 ≤ r < 0.5), and weak relationship (r < 0.3) [32]. Independent-samples T-test was used to analyze the differences between the dominant and non-dominant leg in the YBT (ANT, PL, PM, COM) SLHD tests. The linear regression model was used to estimate the impact of the YBT dominant results (directions COM, PL, PM) on the SLHD dominant leg. 

Statistical data were processed with the Statistica software (Statistica 12). Significance was set a priori at the *p* < 0.05 level.

## 3. Results

### 3.1. The Characteristics and Association between Legs in the YBT and SLHD of the Studied Group of Adolescent Rugby Players

The characteristics of the studied group of adolescent rugby players are presented in Table 1.

There was no significant difference between the YBT and SLHD results for the dominant leg and non-dominant leg test (*p* > 0.05), among adolescent amateur rugby players. For this reason, the dominant leg results were used for further statistical analyses of variables derived from single-legged tests. The data in Table 2 show all means and standard deviations of the variables.

### 3.2. The Correlations between the Hop Tests and YBT COMb

**Figure 1:**
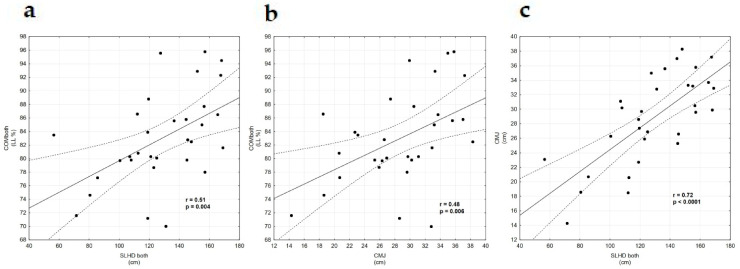
(**a**) Correlation between Y-Balance Test Composite both leg and CMJ. (**b**) Correlation between Y-Balance Test Composite both leg and SLHD both legs. (**c**) Correlation between CMJ and SLHD both legs. Abbreviations: CMJ—Counter Movement Jump, and SLHD—Single Leg Hop for Distance.

There were strong positive correlations between SLHDb and YBT COMb (r = 0.51, *p* = 0.0037) and moderate correlations between the CMJ and YBT COMb test (r = 0.48, *p* = 0.006) among adolescent amateur rugby players. A strong relationship also occurred between SLHDb and CMJ (r = 0.72, *p* < 0.0001); Table 3, Figure 1. However, no significant correlations were found between LESS and the other variables (CMJ, SLHD, YBT) among adolescent amateur rugby players (Table 3).

### 3.3. Linear Regression Model and Correlations for Dominant Leg SLHD and Dominant Leg YBT

**Figure 2:**
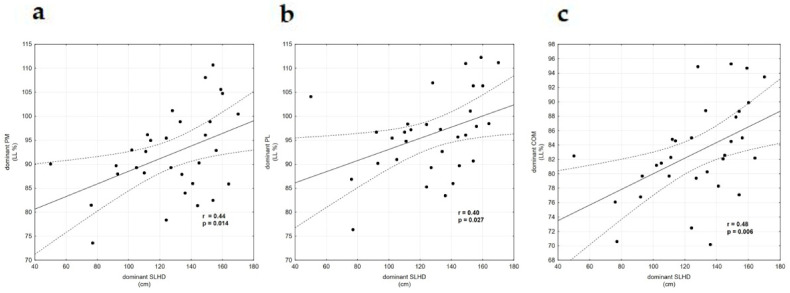
(**a**) Correlation between Y-Balance Test dominant PM and dominant SLHD. (**b**) Correlation between Y-Balance Test dominant PL and dominant SLHD. (**c**) Correlation between Y-Balance Test dominant COM and dominant SLHD. Abbreviations: PM—Posteromedial, SLHD—Single Leg Hop for Distance, PL—Posterolateral, and COM—Composite.

The linear regression models and correlations are illustrated in Table 4 and Figure 2. Pearson’s moderate positive correlation was statistically significant for the dominant leg between the SLHD and YBT PM (r = 0.44), PL (r = 0.40), and Composite (r = 0.48), but not for ANT (r = 0.32, *p* = 0.73). It was found that the strongest predictor of the Composite YBT dominant was the SLHD dominant (adjusted r2 = 0.20, *p* = 0.006). Thus, the SLHD accounted for 20% of the variation in the Composite YBT.

## 4. Discussion

In this study, we examined the relationship between dynamic balance, errors in the landing pattern, and jump abilities during the countermovement jump and the horizontal single-leg jump among young rugby players. A major finding was that the dynamic balance (YBT Composite) was significantly correlated with the power of the lower limbs, as measured by the CMJ (r = 0.48) and SLHD (r = 0.51) tests. The above positive correlations prove that the participants with better dynamic balance have lower limb power.

Moreover, there is evidence from other research demonstrating interventions to increase strength after balance training, and vice versa, balance training to increase balance [20,33,34].

Similar results are presented by Booysen et al., 2015 demonstrating the relationship between power and dynamic balance using the same Y-Balance and CMJ tests. Significant moderate correlations were observed in the adult university and professional football group, but only for the use of the non-dominant leg for the stance in both tests [13]. By measuring the same characteristics, but with different test methods, Erkmen et al. demonstrated a negative correlation between balance (Balance Error Score System) and the power of the lower extremities (Vertical Jump Test) [19]. Contrary to our results, Granacher and Gollhofer 2011 found no relationship between the CMJ test results and static and reactive balance in adolescent students [20]. The reason for the different results of Granacher and Gollhofer and our research can be found in the various research methods used. In our study, we used the specific Y-balance Test for dynamic balance assessment, while Granacher and Gollhofer used the center of pressure (COP) displacements on the balance platform assessment method measured in millimeters.

Moreover, we observed a relationship between the measurements of the CMJ and SLHD in the group of adolescent rugby players. This predicted result is probably due to the involvement of the muscle power responsible for performing the maximum jumps in both tests. The observed strong correlation (r = 0.72) between the vertical jump and the horizontal jump is similar to the results described in the literature [35,36,37]. 

However, no statistically significant correlations were detected between power measurements, dynamic balance, and jump-landing movement pattern errors, among our adolescent amateur rugby players. This suggests that the errors in the biomechanics of the jump-landing are not related to the balance and power of the lower limbs in our study. The lack of significant correlations of the LESS test in adolescent amateur rugby players could be due to the specificity of the LESS test (a large number of variances, most items on a dichotomous scale), which might make it difficult to compare the tests with each other. The lack of a significant relationship between the LESS test and the composite reach YBT of both lower limbs also occurred in the study by de la Motte et al., 2016, where research showed no correlation in young adult male military applicants. In contrast, however, there was a negative correlation between these variables in the female population. Unfortunately, these results cannot be directly compared with ours, due to the modification of the LESS scoring rubric (adding additional test error evaluation points) [38].

Our study did not demonstrate any differences between the dominant leg and nondominant leg in unilateral SLHD and YBT tests, among adolescent amateur rugby players. This result suggests no significant asymmetry between the rugby players’ legs. This allowed only the dominant limb to be selected for analysis. Considering the results for the dominant leg, significant moderate positive correlations were found between the SLHD dominant and all directions of the YBT test, except for the ANT direction. Linear regression analysis showed that the composite direction of the dominant leg was the strongest predictor responsible for 20% of the SLHD dominant leg variation.

Correlations between unilateral hop tests and single-leg dynamic balance can be explained by the strategy of developing the ability to maximally develop the strength of the lower limbs. In both tests, the best results require the use of strong ankle, knee, and hip extensor contractions, to withstand high torques and flexor torques during the flexion phase [39,40]. The extensor muscle groups of the hip and knee are responsible for controlling movement, while reaching the maximum distance in all directions of the YBT test, and assists in returning to the stable starting position of the test [13]. Moreover, it is worth noting that to pass the SLHD test, the participant is required to maintain a stable position (not lose balance) for at least 2 s. Such a rule undoubtedly requires participants to have the ability of dynamic equilibrium immediately after landing in a position similar to squat one-leg, which is observed during the YBT tests.

To our knowledge, this is the first study examining the relationship between the presented tests among youth amateur rugby players. Participants in our study obtained a composite reach score of 83% for both the dominant and non-dominant leg (normalized to the length of the lower limbs). There is evidence that a composite on the Star Excursion Balance Test (a precursor of the YBT) score of less than 94% is associated with an increased risk of injury [41]. In addition, the result of the jump-landing task Landing Error Score System in our study present an average result of 5.5 points, which indicates “poor” landing techniques. According to Padua et al., 2015, a LESS test score of 5 or more could increase the risk of ACL injury for elite youth football players [22]. Additionally, among the elite female basketball players, Šiupšinskas et al., 2019 concluded from their research that the “poor” result might indicate a higher risk of injury [42]. The above evidence might suggest that the players in this study might be at risk of an injury. Moreover, CMJ scores were significantly lower than in the adolescent (16.9 ± 0.4 years) rugby union players from the UK (33.80 ± 5.20 cm) [43] and adolescents (15.3 ± 0.65 years) in the Italian national rugby team level (32.6 ± 5.3 cm) [44]. For the above reasons, extrapolation to other sport groups might be the limit of our study and their comparison should be carried out with a certain degree of caution. Furthermore, no kinematic knee flexion assessment was used to standardize flexion during the CMJ and SLHD test, which could be regarded as confounders. The small number of study participants was also considered a limitation. Therefore, the study was marked as “A Preliminary Study”. Future research should be carried out on a larger number of populations, at different levels of rugby experience.

Significant relationships between dynamic balance and power of the lower limbs in our study indicate that they are dependent on each other. These results can be used in future research on both the implementation of preventive training and the strengthening of individual characteristics of the performance of the sport. Thereby affecting the reduction and complexity of training interventions. Importantly, all limitations of the study should be considered for practical implications.

## 5. Conclusions

The findings of the current study demonstrated significant moderate to strong positive correlation between dynamic balance, and the power of lower extremities in adolescent rugby players. All Y-Balance Test directions, except the anterior, are moderate predictors of the horizontal jump in the Single Leg Hop for Distance Test. In addition, the results of this study suggest that incorrect jump-landing task patterns in the LESS test do not have a significant relationship with the dynamic balance and power of the lower extremities.

The relationship between power and balance can have several practical implications. First, the variables that are dependent on each other, can help in the assessment of risk of injury in young athletes. Second, it will help in designing preventive injury interventions and increasing sports performance. For example, by reducing the volume, amount, and time of exercise by focusing more accurately, e.g., on lower limb power training, thus, increasing the results of the dynamic balance in young athletes.

## Figures and Tables

**Table 1:** Anthropometric characteristics, rugby experience, position, and dominant leg.

	Mean	Std Dev
Age (years)	14.3	1.6
Height (cm)	171.4	9.7
Body mass (kg)	80	26
BMI (m/kg)	27	7
Rugby training experience (years)	4	3
Dominant leg	right = 27	
	left = 4	
Position on the pitch	Forward =18	
	Backs = 13	

Abbreviations: BMI—Body Mass Index.

**Table 2:** Variables and association between dominant and non-dominant legs in YBT and SLHD. Results for dominant and non-dominant leg in the YBT and SLHD, and LESS and CMJ tests.

Variable		Dominant	Nondominant	*p*-Value *
Y-Balance Test LL%	ANT	60.63 ± 7.21	59.40 ± 7.58	0.51
	PL	96.27 ± 8.55	95.08 ± 8.90	0.59
	PM	92.13 ± 8.78	94.53 ± 11.55	0.36
	COM	82.99 ± 6.62	82.99 ± 7.35	0.99
SLHD (cm)		127.26 ± 29.25	129.87 ± 30.73	0.73
Y-Balance Test LL% mean both legs	COMb	83.02 ± 6.80		
SLHD mean both legs (cm)	SLHDb	128.59 ± 29.53		
LESS (points)		5.52 ± 1.52		
CMJ (cm)		28.75 ± 6.14		

Abbreviations: LL%—Limb Length%, SLHD—Single Leg Hop for Distance, LESS—Landing Error Score System, CMJ—Counter Movement Jump, *p*-value *—T-test for Independent Samples.

**Table 3:** Pearson’s correlations between hop tests and YBT COMb.

	CMJ	SLHD	LESS
CMJ			
SLHDb	0.72 *		
LESS	0.06	−0.1	
COMb	0.48 *	0.51 *	0.19

Abbreviations: SLHD—Single Leg Hop for Distance, LESS—Landing Error Score System, CMJ—Counter Movement Jump, * *p* < 0.05.

**Table 4:** Linear regression model with Pearson’s correlation between the dominant leg SLHD and dominant leg YBT.

	R	Adj r2	*p*-Value	Strength
SLHD				
Y-Balance Test				
ANT	0.32	0.07	0.73	Moderate
PL	0.4	0.13	0.027 *	Moderate
PM	0.44	0.16	0.014 *	Moderate
COM	0.48	0.2	0.006 *	Moderate

Abbreviations: ANT—Anterior, PL—Posterolateral, PM—Posteromedial, and COM—Composite, * *p* < 0.05.

## Data Availability

The data presented in this study are available on request from the corresponding author.
